# Computing foaming flows across scales: From breaking waves to microfluidics

**DOI:** 10.1126/sciadv.abm0590

**Published:** 2022-02-02

**Authors:** Petr Karnakov, Sergey Litvinov, Petros Koumoutsakos

**Affiliations:** 1 Computational Science and Engineering Laboratory, ETH Zurich, Clausiusstrasse 33, Zurich CH-8092, Switzerland.; 2 Computational Science and Engineering Laboratory, School of Engineering and Applied Sciences, Harvard University, 29 Oxford Street, Cambridge, MA 02138, USA.

## Abstract

Crashing ocean waves, cappuccino froths, and microfluidic bubble crystals are examples of foamy flows. Foamy flows are critical in numerous natural and industrial processes and remain notoriously difficult to compute as they involve coupled, multiscale physical processes. Computations need to resolve the interactions of the bubbles separated by stable thin liquid films. We present the multilayer volume-of-fluid method (Multi-VOF) that advances the state of the art in simulation capabilities of foamy flows. The method introduces a scheme to handle multiple bubbles that do not coalesce. Multi-VOF is verified and validated with experimental results and complemented with open-source software. We demonstrate capturing of crystalline structures of bubbles in realistic microfluidics devices and foamy flows involving tens of thousands of bubbles in a waterfall. The present technique extends the classical volume-of-fluid methodology and allows for large-scale predictive simulations of flows with multiple interfaces.

## Teaser

Numerical method Multi-VOF describes flows with thousands of noncoalescing bubbles that turn into foam

## INTRODUCTION

Flows with bubbles and drops are central to new products and technologies in areas ranging from food to cosmetics and drug delivery through precision microfluidics for emulsions and foams (*1*–*3*). Drainage and rupture of the liquid film separating the bubbles leads to their coalescence. Surfactants (*4*) and other impurities, such as electrolytes (*5*), in the liquid can delay or prevent coalescence. Bubbles in clean liquids can also collide without coalescence if the film drainage time is sufficiently large (*6*). Foams are composed of many bubbles separated by stable liquid films. Foams are structural elements of insect, fish, and frog nests and central to numerous industrial processes and medicine (*7*–*9*).

The simulation of foamy flows, involving noncoalescing bubbles, presents a number of formidable challenges in addition to those associated with resolving the bubble interactions with the fluid flow and the solid boundaries (*10*). In foams, the thickness of the film is usually several orders of magnitude smaller than the size of bubbles (*11*). In the limit of zero film thickness, the evolution of (dry) foams can be predicted using Lagrangian techniques that track the surfaces between bubbles with multiple junctions (*12*). State-of-the-art techniques involve Eulerian methods such as the Voronoi implicit interface method (VIIM) (*13*, *14*) that uses a single level-set function for all interfaces. More recently, methods such as the lattice Boltzmann (LB) (*15*) have captured the effect of thin films using an empirical collision potential but resort to mesoscale models with limited density ratio and artificial compressibility. In simulations of bubble collisions in turbulent flows (*16*), a short-range repulsive force between bubbles has been used to increase the surface tension near the contact. However, this can only remedy rapid collisions but not account for stable multibubble structures with thin films as in foams.

The classical volume-of-fluid (VOF) methodology (*17*, *18*) has found widespread success in simulations of engineering flows involving interfaces. However, VOF leads to spurious coalescence of bubbles that approach each other at a distance below one computational cell. This spurious coalescence can be prevented by introducing distinct volume fraction fields for each bubble in the multimarker VOF (*19*) or distinct functions in level-set methods (*20*). This prevention of coalescence has a computational cost that is proportional to the number of bubbles in the simulation [i.e., O(*N*_bubbles_ *N*_cells_)], and it is prohibitive for systems with a few hundred bubbles even in today’s computer architectures. Furthermore, since each distinct volume fraction field corresponds to only one bubble, keeping track of all volume fraction fields is redundant. The local approach (*21*) assumes that the computation is distributed among many processors and only keeps track of nonempty fields on each processor. Therefore, the number of required fields scales as the number of bubbles per processor, which is still not suitable for cases with many small bubbles. We propose a method to store multiple fields in a compact way so that the number of the necessary scalar fields is constant, independent of the number of bubbles in the simulation (see Materials and Methods). The method considers multiple layers of volume fraction fields and assigns colors to bubbles to distinguish them. A similar approach with colors has been formulated in the context of LB methods (*22*, *23*). This technique is combined with VOF to simulate flows with noncoalescing bubbles. The proposed multilayer VOF method (Multi-VOF) overcomes the abovementioned challenges and allows for predictive simulations of flows involving thousands of interacting and noncoalescing bubbles. Multi-VOF requires only a fixed number of fields, and its computational cost is independent of the number of bubbles in the simulation.

## RESULTS

### Constrained mean curvature flow, comparison with VIIM

**Fig. 1.**
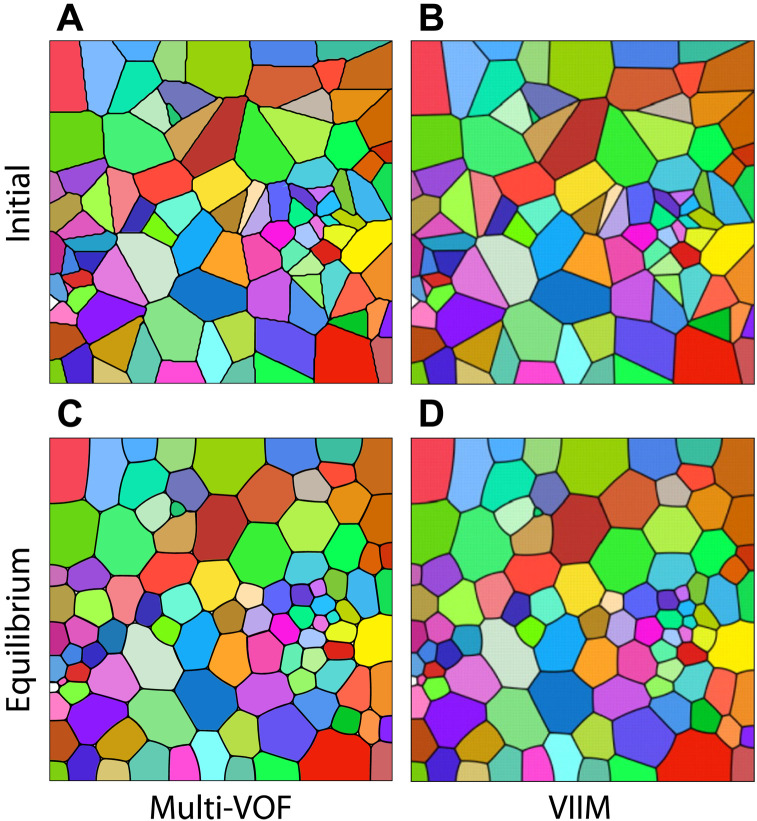
Constrained mean curvature flow. (**A** and **B**) Initial configurations of components representing a Voronoi diagram. (**C** and **D**) Equilibrium configurations obtained by Multi-VOF (C) and VIIM (*13*) (D) on the same mesh of 256^2^ cells. Interfaces at triple junctions form 120° angles.

The capabilities of Multi-VOF are first assessed in the limiting case of fluid flows with surface tension but no inertia. In this case, the governing equations (see Materials and Methods) simplify to a single equation for the velocity field ***u*** = κ***n***δ_S_ − ∇ *p*, where κ is the interface curvature, ***n*** is the interface normal, δ_S_ is the delta function on the interface, and the scalar field *p*(***x***, *t*) is used to ensure that the velocity field is divergence-free (∇ · ***u*** = 0). We use this velocity field with the advection equation to compare Multi-VOF with the pioneering work (*13*). The initial conditions are adopted from (*13*) and represent a Voronoi diagram of a randomly chosen set of 100 points with homogeneous Neumann boundary conditions for the volume fraction fields. Initially, the interfaces are straight lines and form multiple junctions at arbitrary angles. As time evolves, only triple junctions remain, and the angles between the lines approach 120°. In Fig. 1, we compare the solution by our method and the VIIM (*13*). We note that the study of Saye and Sethian (*13*) imposes different constraints on the velocity field as it penalizes the change of area of components, while we find a divergence-free velocity field throughout the domain. For Multi-VOF, dry foams are a limiting case since there is no special treatment of triple junctions leading to the formation of small voids near the junctions. Penalization techniques that add source terms proportional to the volume of voids can reduce the voids. Nevertheless, the results of Multi-VOF and VIIM agree for the same mesh size.

### Microfluidic crystals

Bubbles and droplets in microfluidic devices organize into lattices called microfluidic crystals (*24*). They serve as prototypes of foam structures, compartments for chemical reactions, and parts in production of metamaterials (*25*). A recent study (*15*) applied a mesoscale LB model to capture these flow structures. The LB model includes a short-range forcing term that describes the combined effect of surface tension and near-contact interactions to prevent coalescence. The authors compare their results with experimental data (*25*) on foams of air bubbles in water. However, because of the limited density ratio of the LB approach, their mesoscale model is instead tuned for water drops embedded in oil.

Using the Multi-VOF, we aim to reproduce the experimental study (*26*) on the formation of microfluidic crystals of bubbles in water. Limited by numerical stability and computational cost, we use lower values of the density ratio, surface tension, viscosity, and the channel length. The device is based on a flow-focusing geometry (*27*) and consists in a planar network of rectangular ducts of a height *H* with three inlets. The gas is injected from one inlet at a fixed pressure *P*_g_ relative to the outlet pressure, and the liquid comes through the other two at a total flow rate *Q*_l_. The gas enters the channel through a contracting duct that ends with an orifice of a width *W*_0_ expanding into the collection channel. Walls of the channel are no-slip boundaries. Parameters of the simulation are given in table S1.

Bubbles in these devices are generated by the breakup of the air thread in the inlet channel. The period of breakup is determined by the liquid flow rate and not by capillary time scales despite an apparent similarity to the Rayleigh-Plateau instability (*27*). In simulations, we forcibly separate the air thread at a regular interval *T*_b_. Unless stated otherwise, the period of breakup is equal to $T_{b}=0.5\;W_{0}^{2}H/Q_{l}$, estimating the time it takes for the liquid to fill the volume of a cavity forming right before the breakup.

**Fig. 2.**
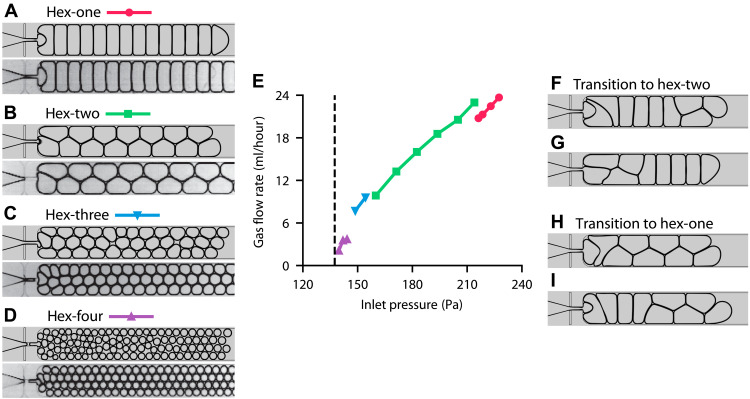
Microfluidic crystals. (**A** to **D**) Crystalline structures of bubbles from simulations compared to experimental images (*26*). Hex-one [*P*_g_ = 216 Pa (A)], hex-two [*P*_g_ = 194 Pa (B)], hex-three [*P*_g_ = 149 Pa (C)], and hex-four [*P*_g_ = 140 Pa (D)]. Reprinted images with permission from (*26*). Copyright 2009 by the American Physical Society. (**E**) Gas flow rate as a function of the inlet gas pressure. The flow starts once the pressure exceeds the capillary threshold (dashed line). (**F** to **I**) Spontaneous transitions between hex-one and hex-two with *L* = 5.3 mm and *P*_g_ = 225 Pa. Snapshots are taken at times 443 ms (F), 460 ms (G), 512 ms (H), and 529 ms (I).

To obtain various crystalline structures, we vary the inlet gas pressure *P*_g_. The gas flow rate *Q*_g_ as a function of pressure is plotted in Fig. 2. The gas flow starts as soon as the pressure exceeds the capillary threshold estimated as *P*_cap_ = σ(1/*H* + 1/*W*_0_). Raising the pressure enhances the gas flow rate *Q*_g_ and, since the breakup period *T*_b_ is kept constant, increases the size of bubbles. Each simulation with a given *P*_g_ is advanced until equilibration, the evolution of the bubble volume for selected values of *P*_g_ is shown in fig. S13. Closely packed bubbles form 120° angles at triple junctions, and junctions at the walls are 90°. Depending on the size, the bubbles organize into regular structures or flowing crystals. The structures are named by the number of bubbles that fit in the channel width (*26*): hex-one, hex-two, hex-three, and so on. Examples of the structures together with experimental images are shown in Fig. 2 and movies S1 to S4. Stability of each structure is dictated by the corresponding value of the surface energy. Smaller bubbles transition to higher-order structures.

The dissipation in foam is proportional to its wetting perimeter, i.e., the length of the menisci that confine the liquid between the interface and the channel walls (*28*). Hex-one bubbles dissipate more than hex-two bubbles and correspond to a slower flow (*26*). Our simulations capture this effect as seen from Fig. 2 and movie S5. If the structure remains the same, then increasing the pressure leads to a faster flow. Conversely, if the structure changes, then increasing the pressure may decrease the flow rate. For example, increasing *P*_g_ from 205 to 216 Pa reduces the flow rate as the flow transitions from hex-two to hex-one.

One type of behavior observed near these transitions is a bubbling oscillator (*29*). It is based on the interplay between the stability and dissipation of hex-one and hex-two structures. As the channel fills with hex-one bubbles, the flow rate decreases due to growing dissipation. Bubbles entering the channel become smaller such that the hex-one structure is no longer stable. The flow transitions to hex-two and accelerates again. The process repeats indefinitely. In our simulations, we obtain such an oscillator with *L* = 5.3 mm, $T_{b}=0.46\;W_{0}^{2}H/Q_{l}$, and the inlet pressure *P*_g_ = 225 Pa. The bubble volume plotted in fig. S13 oscillates in time. Examples of the transitions between hex-one and hex-two are shown in Fig. 2.

### Bidisperse foam generation

A recently demonstrated microfluidic device (*30*) makes use of bubble-bubble pinch-off to generate bidisperse foams. Here, we reproduce its operation numerically. The device represents a planar network 60 μm high where a narrow channel expands with 45° walls to a collection channel. Bubbles are generated periodically at an interval of 111 μs to maintain the volume fraction of gas at 0.645. At each cycle, the bubble is inserted in the narrow channel by replacing the liquid with gas in a part of the channel. Parameters of the simulation are summarized in table S2. Walls of the channel are no-slip boundaries.

**Fig. 3.**
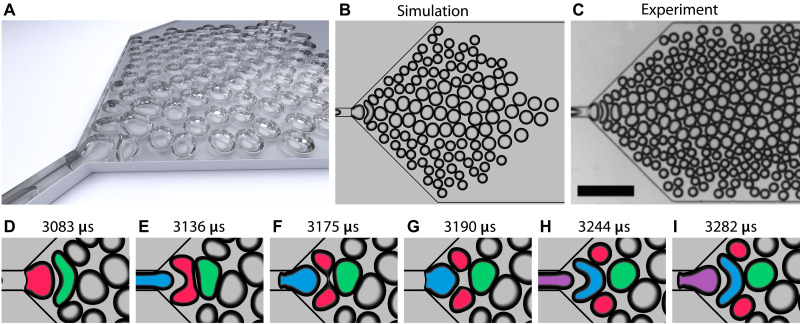
Bidisperse foam generation. (**A**) Overall view of the microfluidic device for generation of bidisperse foams at *t* = 8749 μs. (**B**) Snapshot from the simulation at *t* = 8872 μs. (**C**) Experimental image from (*30*). Scale bar, 500 μm. Republished with permission of the Royal Society of Chemistry; permission conveyed through Copyright Clearance Center Inc. (**D** to **I**) Alternation between bubble-bubble pinch-off and elongation without breakup. Snapshots at *t* = 3083, 3136, 3175, 3190, 3244, and 3282 μs, respectively. Colors highlight the same bubbles in all snapshots. Split bubble (red), wall bubble (green), and intact bubble (blue).

The overall view of the device is shown in Fig. 3A and movie S6. The snapshot from the simulation in Fig. 3B compared to the experimental image (Fig. 3d therein) (*30*) indicates a good agreement with the experimental data since both have similar shapes and positions of the split and intact bubbles.

Bubbles entering the expansion alternate between two types of behavior illustrated in Fig. 3 (D to I). They either split into smaller bubbles or remain intact. For example, one bubble (Fig. 3D) enters the expansion, elongates under the shear stress (Fig. 3E) from the liquid flow, and splits into two daughter bubbles (Fig. 3F). The wall bubble confines the liquid flow. The two daughter bubbles then migrate sideways, leaving a gap of liquid between the wall bubble (Fig. 3G) and the next incoming bubble, which only elongates without breakup and eventually restores its shape. Alternation of these two regimes drives the generation of bidisperse foams.

**Fig. 4.**
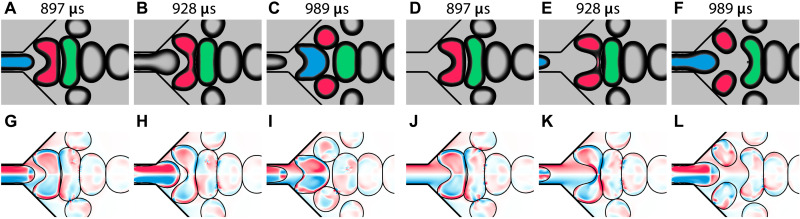
Effect of delaying the pincher bubble. Standard case (**A** to **C** and **G** to **I**) and case with delayed pincher bubble (**D** to **F** and **J** to **L**) at various times. (A to F) Bubble surfaces with the highlighted pincher bubble (blue), split bubble (red), and wall bubble (green). (G to L) Component of vorticity normal to the view plane (red for counterclockwise and blue for clockwise). Delaying the pincher bubble does not prevent breakup.

Another part of the pinch-off process is the pincher bubble upstream of the split bubble (blue in Fig. 3E and purple in Fig. 3H). One question arising here is whether the pincher bubble actually causes the breakup. The explanation given in the experimental study (*30*) suggests that the pincher bubble increases the flow confinement and the corresponding shear stresses on the split bubble, leading to breakup. To verify this, we compare two simulations: one with a normal pinch-off event and one where the pincher bubble is delayed by 60 μs. Both simulations were done with the length of the collection channel set to *L* = 0 μm to reduce the computational cost, still leaving a sufficient separation from the outlet. Figure 4 shows that the pincher bubble does not trigger the pinch-off event. Delaying the pincher bubble does not change the flow of the liquid near the split bubble (Fig. 4, H and K). This indicates that the pinch-off is triggered by the wall bubble downstream rather than the pincher bubble upstream.

### Clustering of bubbles

Clustering of bubbles floating on the surface of water is an example of self-assembly known as the Cheerios effect (*31*), named after the observation that breakfast cereals floating in milk often clamp together. A bubble floating on the surface creates an elevation attracting other bubbles due to buoyancy. Many floating bubbles are hence attracted to each other and form clusters.

**Fig. 5.**
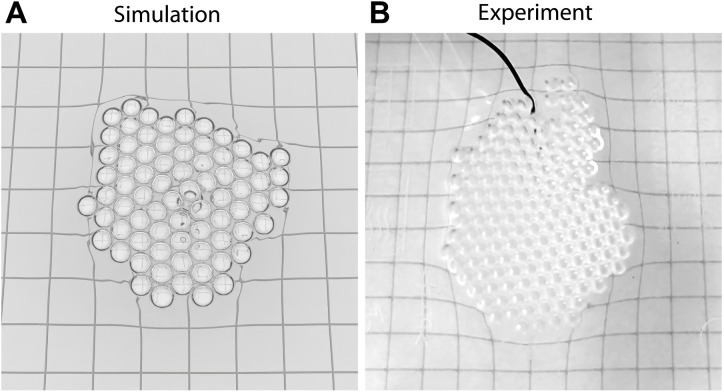
Clustering of bubbles floating in water. (**A**) Simulation using Multi-VOF. (**B**) Experiment in soapy water. The size of square cells is 4 mm.

Figure 5 shows clusters of bubbles obtained numerically and experimentally. In the experimental setup, a large tank is filled halfway with tap water and a common detergent. One end of a tube with an inner diameter of about 0.3 mm is submerged into water, and the other end is connected to a syringe filled with air. The plunger is then abruptly pushed until bubbles start to appear. This generates bubbles of about 2 mm in diameter. To visualize the deformation of the surface, we cover the bottom of the tank with a patterned sheet. In the simulation, spherical bubbles are generated at the bottom at regular intervals. Parameters are in table S3. Both in the experiment and the simulation, bubbles floating on the surface form clusters and organize in a hexagonal lattice. Movie S7 shows the simulation results.

### Foaming waterfall

**Fig. 6.**
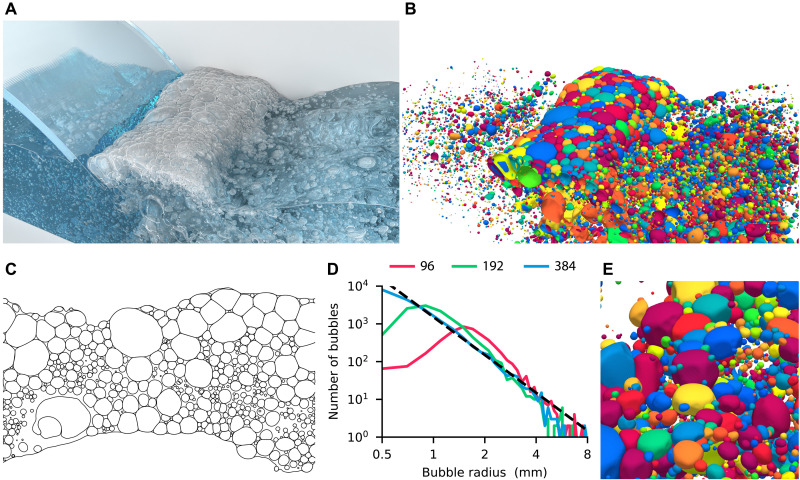
Foaming waterfall. (**A**) Overall view of the interface at *t* = 1.2 s. (**B**) Same view showing bubbles under the surface with arbitrary colors. (**C**) Horizontal cross section of the interface at *t* = 1.2 s. Clusters of bubbles on the surface show characteristic features of foam: Bubbles are separated by thin membranes (lamellae) with multiple junctions (Plateau borders). (**D**) Histogram showing the number of bubbles *N*(*r*) with the equivalent radius in the range *r* ± 0.1 mm at time *t* = 1.2 s. Results for different mesh sizes of 96 (red), 192 (green), and 384 (blue) cells in the height compared to scaling law *N*(*r*) ∝ *r*^−10/3^ (dashed line) from (*33*). (**E**) Close-up of (B) with half of the bubbles removed.

Natural surfactants in sea water can suppress coalescence as well. Oceans are covered with foam generated by breaking waves. The following application is an example of these flows in the limiting case without coalescence. A 100-mm-high rectangular tank is filled halfway with water. A waterfall enters the tank at a given velocity. Table S4 lists the simulation parameters. Results of the simulation are in Fig. 6 with additional snapshots in fig. S16 and movie S8. On the finest mesh consisting of 768 × 384 × 384 cells, the simulation took 24 hours on 13,824 cores of the Piz Daint supercomputer equipped with 12-core Intel Xeon E5-2690 v3 processors. Two major mechanisms of air entrainment (*32*) are observed in this simulation: entrapment of a tube of air when the sheet of water impacts the surface and entrainment around the impact site as the waterfall drags air into the water. The entrained bubbles rise to the surface and create a layer of foam. As seen from the horizontal cross section of the foam in Fig. 6, the bubbles are separated by thin membranes (lamellae) that form multiple junctions (Plateau borders) at angles approaching 120°.

The distribution of the bubble size in Fig. 6 matches a scaling law *N*(*r*) ∝ *r*^−10/3^ from (*33*), where *N*(*r*) is the number of bubbles of radii in the range *r* ± 0.1 mm. The model stems from the assumption that the inflow of air per unit volume is constant, and the number of bubbles depends only on the turbulent dissipation rate and the bubble radius. This scaling law is commonly observed for bubbles generated by breaking waves and has been reported in experimental (*34*) and numerical (*35*) studies.

## DISCUSSION

The Multi-VOF can simulate flows with many bubbles and drops that do not coalesce. It represents many bubbles with a fixed number of volume fraction fields and assigns colors to bubbles to distinguish them. An additional technique for interface regularization, described in the Supplementary Materials, based on forward-backward advection improves the accuracy of the advection scheme. The presented applications show that the method can reproduce experiments on generation of foam in microfluidic devices and clustering of bubbles floating in water.

The proposed methodology advances the state of the art in simulations of flows with multiple interfaces in the following categories:

1) Efficiency: The method uses a fixed number of volume fraction fields on an Eulerian mesh. The computational complexity of the advection algorithm is linear with the number of cells and does not depend on the number of bubbles.

2) Compatibility with existing methods: The method is compatible with existing stencil-based methods for interface capturing and curvature estimation, including the popular VOF and level-set methods. For dry foams, the method can recover the results of VIIM at comparable resolutions although may lack asymptotic convergence due to constant errors at triple junctions.

3) Capturing topology changes and multiple junctions: Breakup and coalescence of bubbles including thin liquid films and triple lines are captured without special treatment.

4) High performance implementation: The method only involves stencil operations and is readily integrated in high performance software for structured grids.

We believe that Multi-VOF opens new horizons for simulating a wide variety of flows from the micro to the macroscale, including wet foams, turbulent flows with bubbles, suspensions and emulsions in microfluidics. Moreover, the efficiency of the code allows for extensive studies in control and optimization of bubbly flows.

### Limitations

The method only describes the complete prevention of coalescence assuming that the thin liquid films between bubbles never rupture. This corresponds to the stabilization of liquid films by surfactants. However, effects of surfactants on surface tension are not considered. Cases when the liquid films undergo rupture would require an empirical coalescence criterion. As with the original multimarker approach (*19*), the effects of viscous flow in thin liquid films are not described. They are less significant if the viscosity ratio is close to unity, e.g., the drop impact in fig. S12, and otherwise can be considered in a multiscale model including transport equations on surfaces of bubbles (*14*). Since bubbles are distinguished as connected components of the volume fraction field, the method does not prevent coalescence if a deformed bubble folds back onto itself. Two small bubbles can penetrate each other and form concentric configurations when their radius is comparable to one computational cell. The method can be improved by adopting a semi-implicit discretization of the surface tension force (*36*).

In the study of microfluidic crystals, additional modeling is required for the generation of bubbles. In simulations, we forcibly separate the air thread at regular intervals. Otherwise, the gas thread remains continuous unless the inlet pressure is sufficiently low. These continuous regimes are observed experimentally under certain conditions (*37*) but not in the experimental study of interest (*26*). One explanation for this discrepancy is the effect of wetting (*37*) that may narrow the gap between the liquid-gas interface and the channel walls since the static contact angle of polydimethylsiloxane is 104° (*38*), while our model assumes 180°. Another possibility is that the viscous flow of the liquid upstream of the orifice is not sufficiently resolved in the simulations.

## MATERIALS AND METHODS

### Multilayer fields

Consider a discrete domain Ω consisting of cells *c* ∈ Ω, where the number of cells is ∣Ω∣= *N*_cells_. A conventional cell field ϕ : *c* ↦ ϕ*_c_* is a mapping from cell *c* to a value. A cell-color field $\hat{\phi }:(c,q)\mapsto {\hat{\phi }}_{c}(q)$ is a mapping from cell *c* and color *q* ∈ ℝ to a value. Overlapping bubbles can be represented by a single cell-color field if each bubble is assigned a unique color. The restriction operation ${\hat{\phi }|}_{q}$ constructs a conventional field ${\hat{\phi }|}_{q}:c\mapsto {\hat{\phi }}_{c}(q)$ from a cell-color field $\hat{\phi }$ given a color *q*. Using this operation, any standard routine, such as computing the normals or solving the advection equation, can be applied to a cell-color field by individually selecting all possible colors. To store a cell-color field $\hat{\phi }$, we use a sequence of conventional fields for values and colors separately. Assume that any cell contains at most *L* bubbles and their shapes are represented by the cell-color volume fraction field $\hat{\alpha }$. The colors are stored in fields $q^{l}:c\mapsto q_{c}^{l},l=1,\ldots ,L$ defined in each cell *c* as

$$q_{c}^{l}=\{\begin{array}{cc} q_{l}, & 1\leq l\leq L^{\prime }\\ q_{\text{none}}, & \text{otherwise} \end{array}$$

where {*q*_1_, …, *q*_*L*^′^ ≤ *L*_} are all colors for which ${\hat{\alpha }}_{c}(q)>0$ and *q*_none_ is a distinguished none color (e.g., *q*_none_ ≔ − 1). The corresponding values are stored in fields $\phi^{l}:c\mapsto \phi_{c}^{l},l=1,\ldots ,L$

$$\phi^{l}=\{\begin{array}{cc} \hat{\phi }(q_{c}^{l}), & q_{c}^{l}\neq q_{\text{none}}\\ \text{undefined}, & \text{otherwise} \end{array}$$

**Fig. 7.**
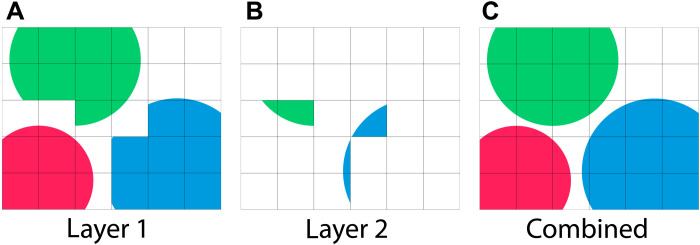
Two-layer volume fraction field representing three bubbles. Individual layers (**A** and **B**) contain at most one bubble per cell, while the combined field (**C**) describes overlapping interfaces.

The pairs (ϕ*^l^*, *q^l^*) are referred to as layers and the sequences (ϕ^1^, …, ϕ*^L^*) and (*q*^1^, …, *q^L^*) constitute a multilayer field. The order in which the colors are stored is insignificant, i.e., all sequences $\left(q_{c}^{1},\ldots ,q_{c}^{L}\right)$ and $\left(\phi_{c}^{1},\ldots ,\phi_{c}^{L}\right)$ are equivalent up to mutual permutation. Figure 7 illustrates two layers that describe three overlapping bubbles.

### Advection

By constructing conventional fields from a cell-color field, we can apply standard stencil-based algorithms to cell-color fields. One such algorithm, the PLIC (piecewise linear interface characterization) method (*39*) for advection, is described in the following. The PLIC method solves the advection equation $\frac{\partial \alpha }{\partial t}+(u\cdot \nabla )\;\alpha =0$ given a velocity field ***u***. As the name stands, it performs a piecewise linear reconstruction by replacing the interface in each cell with a plane. Apart from the volume fraction field, it involves normals and plane constants. The normals ***n*** : Ω → ℝ^3^ are estimated from the volume fractions using the mixed Youngs-centered scheme (*40*), and the plane constants are computed from the normals and volume fractions using explicit formulas (*41*). The fluid volume is reconstructed in each cell with a polyhedron formed by cutting the cell with the plane. The fluxes are then computed by advecting the polyhedrons according to the given velocity (*40*, *42*). The discretization uses directional splitting, and a step in one direction can be schematically written in terms of discrete operators N and A: $n_{c}=N(S^{c}[\alpha ])$ and $\alpha_{c}^{\text{new}}=A(S^{c}[\alpha ],S^{c}[n])$, where *S^c^* = (*c*_1_, …, *c*_27_) is the sequence of cells in the 3 × 3 × 3 stencil centered at *c* and *S^c^*[ϕ] = (ϕ_*c*_1__, …, ϕ_*c*_27__) are the corresponding values of a field ϕ. To apply this method to a cell-color field, the same procedure is repeated for all colors: ${\hat{n}}_{c}(q)=N(S^{c}[{\hat{\alpha }|}_{q}])$ and ${\hat{\alpha }}_{c}^{\text{new}}(q)=A(S^{c}[{\hat{\alpha }|}_{q}],S^{c}[{\hat{n}|}_{q}])$. In terms of multilayer fields *q^l^*, α*^l^*, and ***n****^l^*, the normals are computed with the following algorithm

**for** c ∈ Ω,*l* = 1, …, *L* **do**

    **if**  $q_{c}^{l}\neq q_{\text{none}}$ **then**

        **for** *i* = 1, …, 27 **do**

            $c^{\prime }\leftarrow S_{i}^{c}$

            ${\bar{\alpha }}_{i}\leftarrow 0$

            **for** *l*′ = 1, …, *L* **do**

                **if** $q_{c\prime }^{l\prime }=q_{c}^{l}$ **then**

                    ${\bar{\alpha }}_{i}\leftarrow \alpha_{c\prime }^{l\prime }$

                **end if**

            **end for**

        **end for**

        $n_{c}^{l}\leftarrow N(\bar{\alpha })$

    **end if**

**end for**

The total number of operations of this algorithm is O(*L*^2^ *N*_cells_). Before proceeding with advection, the normals are corrected. When *L*′ ≥ 2 interfaces enter one cell, we ensure that their normals are parallel. From the estimated normals (***n***_1_, …, ***n***_*L*^′^_), we compute the average $n_{\text{avg}}=\frac{1}{L\prime }\sum_{l=1}^{L\prime }n_{l}\;\text{sign}\;(n_{l}\cdot n_{1})$ and overwrite the normals as ***n****_l_* ← ***n***_avg_ sign (***n****_l_* · ***n***_avg_). This correction prevents mutual penetration of interfaces.

The advection step is done similarly but includes also new colors found in upwind cells. If the new volume fraction for an upwind cell color is positive, then the color is added to the current cell. Colors corresponding to zero volume fractions are removed. For the number of layers, we find sufficient *L* = 4 based on the case of close packing of rising bubbles in fig. S11.

### Connected component labeling

Prevention of coalescence requires that all bubbles have unique colors. These unique colors can be assigned from initial conditions. For instance, using the indices of bubbles as colors. If the set of bubbles remains the same, then the colors remain unique throughout the simulation. However, new colors are needed for injected bubbles or bubbles formed during breakups. To detect breakups and assign unique colors to all bubbles, we use connected component labeling.

Starting with the old color fields *q^l^* and volume fraction fields α*^l^*, we construct new color fields ${\tilde{q}}^{l}$ in which all bubbles have unique colors. Different bubbles are identified as connected components in the volume fraction field. Two neighboring cells and layers (*c*, *l*) and (*c*′, *l*′) are connected with an edge if they have positive volume fractions $\alpha_{c}^{l}>0$ and $\alpha_{c\prime }^{l\prime }>0$ and equal colors $q_{c}^{l}=q_{c\prime }^{l\prime }$.

To detect the connected components, we first initialize ${\tilde{q}}_{c}^{l}$ with unique colors for all cells and layers. For instance, using an integer index enumerating all cells and layers (in total, *N*_cells_ *L* colors). Then, we iterate until convergence over all pairs of connected cells and layers choosing the minimal color. The procedure is implemented by the following algorithm

*i* ← 0

**for** *c* ∈ Ω, *l* = 1, …, *L* **do**          ⊳ initialize with unique colors

        ${\tilde{q}}_{c}^{l}\leftarrow i$

        i←i+1

**end for**

**repeat**        ⊳ propagate smaller colors until convergence

            *converged* ← True

            **for** *c* ∈ Ω, *l* = 1, …, *L* **do**

                **if** $q_{c}^{l}\neq q_{\text{none}}\&\alpha_{c}^{l}>0$ **then**

                    **for** *c*′ ∈ *S^c^*, *l*′ = 1, …, *L* **do**

                        **if** $q_{c\prime }^{l\prime }=q_{c}^{l}\&\alpha_{c\prime }^{l\prime }>0\&{\tilde{q}}_{c\prime }^{c\prime }<{\tilde{q}}_{c}^{l}$ **then**

                            ${\tilde{q}}_{c}^{l}\leftarrow {\tilde{q}}_{c\prime }^{l\prime }$

                                *converged* ← False

                            **end if**

                        **end for**

                    **end if**

                **end for**

            **until** *converged*

Figure S1 illustrates the algorithm on a case with one layer and three connected components.

If the domain is decomposed into rectangular blocks for distributed parallelization, then the algorithm is first applied to each block, then the halo cells are exchanged through communication, and the whole procedure is repeated until global convergence. The total number of communication steps is the maximum length of a connected component divided by the block size. To reduce the number of communication steps, we use a heuristic for faster propagation of colors over the domain. The heuristic is executed before each iteration of the main algorithm. For each block, we consider only one corner cell (*c*, *l*) and its neighbors (*c*′, *l*′) and, if they are connected, collect the corresponding pairs of colors ${\tilde{q}}_{c}^{l}$ and ${\tilde{q}}_{c\prime }^{l\prime }$ and transfer them all to one processor. Then, we perform connected component labeling on this set of pairs and find a set of connected components. Last, we broadcast them to other processors and replace each color with the minimal element of its connected component. With this heuristic, large connected components are identified after fewer iterations.

### Two-component incompressible flows

The model of two-component incompressible flows consists of the Navier-Stokes equations for the mixture velocity ***u*** and pressure *p*

$$\begin{array}{c} \nabla \cdot u=0\\ \rho (\frac{\partial u}{\partial t}+(u\cdot \nabla )\;u)=-\nabla p+\nabla \cdot \mu (\nabla u+\nabla u^{T})+f_{\sigma }+\rho g \end{array}$$

and the advection equation for the volume fraction α

$$\frac{\partial \alpha }{\partial t}+(u\cdot \nabla )\;\alpha =0$$

with the mixture density ρ = (1 − α)ρ_1_ + αρ_2_, dynamic viscosity μ = (1 − α)μ_1_ + αμ_2_, surface tension force ***f****_σ_* = σ**κ∇** α, and gravitational acceleration ***g***, where σ is the surface tension and κ is the interface curvature. The mixture flow equations are discretized with the projection method (*43*), and the advection equation is solved using the procedure described in the “Advection” section. The mixture density and viscosity fields are computed from the combined volume fraction field α = min (1, ∑*_q_*α∣*_q_*). The surface tension force is computed by summation over all colors ***f***^σ^ = ∑*_q_*σκ∣*_q_*∇α∣*_q_* and the curvature κ∣*_q_* is estimated from the volume fractions using the method of particles (*44*). We apply an interface regularization technique (see the Supplementary Materials), which does not affect asymptotic convergence or conservation properties but results in smoother interfaces at low resolutions. To simulate flows in complex geometries on a Cartesian mesh, we use the method of embedded boundaries (*45*), which approximates the shape of the body with cut cells.

## Supplementary Materials

### This PDF file includes:

Supplementary Text Figs. S1 to S16 Tables S1 to S5 Legends for movies S1 to S8 References
