# Supplementary material for: Computing foaming flows across scales: From breaking waves to microfluidics
Source: Sci Adv. 2022 Feb 2;8(5):eabm0590. doi: 10.1126/sciadv.abm0590 (PMC8809540; doi:10.1126/sciadv.abm0590)
Supplement: Supplementary file 1 — Supplementary Text Figs. S1 to S16 Tables S1 to S5 Legends for movies S1 to S8 References [file sciadv.abm0590_sm.pdf]

Science Advances
AAAS

Supplementary Materials for

Computing foaming flows across scales: From breaking waves to microfluidics

Petr Karnakov, Sergey Litvinov, Petros Koumoutsakos*

*Corresponding author. Email: petros@seas.harvard.edu

Published 2 February 2022, *Sci. Adv.* **8**, eabm0590 (2022)
DOI: 10.1126/sciadv.abm0590

The PDF file includes:

Supplementary Text
Figs. S1 to S16
Tables S1 to S5
Legends for movies S1 to S8
References

Other Supplementary Material for this manuscript includes the following:

Movies S1 to S8

Supplementary Text

Interface regularization

Volume-of-fluid methods based on geometrical reconstruction, such as PLIC (*39*) employed in this work, offer good approximation properties, ensure boundness and conservation. They sharpen the interface of advected objects and hence reduce the numerical diffusion. However, the sharpening effect depends on the advection velocity. The choice of the frame of reference and the location of advected objects in a nonuniform velocity field affect the solution, which is undesirable. To mitigate this and also improve the accuracy at lower resolutions, we propose a technique for interface regularization based on PLIC. The idea is to apply forward and backward advection with a uniform velocity. In one dimension, the technique consists of two advection sweeps illustrated in Fig. S2. The initial interface (step 1) spans two cells with volume fractions $(0.5, 0.5)$. The forward step moves the interface to the right (step $1/2$) and the volume fractions take values $(0.8, 0.5)$ as one cell fills with liquid from upstream. The backward step moves the interface to the left (step 1) reducing the volume fraction in the other cell, so they reach values $(0.8, 0.2)$. Note that after two steps, the field is again symmetric. In three dimensions, there are six advection sweeps with uniform velocities $\boldsymbol{u} = (U, 0, 0)$, $(-U, 0, 0)$, $(0, U, 0)$, $(0, -U, 0)$, $(0, 0, U)$, and $(0, 0, -U)$. The velocity on boundaries is set to zero. They are repeated after every time step of the simulation. The only parameter of the regularization is the advection velocity defined by the CFL number, which we set to $U\Delta t/h = 0.1$ for all simulations. To reduce the computational cost, the pairs of sweeps in each direction can be spread among every three consecutive time steps leaving only two sweeps per time step.

As noted above, the solution by the standard PLIC method depends on the choice of the frame of reference. Moreover, at low resolutions the method can produce small spurious interface fragments. These effects are evident from the evolution of drops in Couette flow shown in Fig. S3. The domain is a unit cube periodic in two directions and no-slip walls at the bottom and top with imposed velocities of 0 and 1 respectively. The mesh size is N^3 with $N = 8$, 16, and 32. Initially, the drops are spherical of a radius 0.15. The Reynolds number is $\mathrm{Re} = \rho/\mu = 100$, the capillary number is $\mathrm{Ca} = \mu/\sigma = 0.1$, and both components have the same density and viscosity. Since the flow is symmetric, in the exact solution the drops need to be symmetric as well. However, the numerical solution lacks this symmetry since the drops move at different speeds. Solving the problem with the standard method without regularization, we observe that the top (red) and bottom (blue) drops have different shapes and small interface fragments appear near the bottom drop at lower resolutions of $N = 8$ and 16. Enabling the regularization not only removes the spurious fragments but also makes the shapes of the two drops more similar thus reducing their dependence on the frame of reference.

Another possible application of this technique is the regularization of initial conditions. Fig. S4 demonstrates two different fields representing a circle: a stepwise approximation and a mollified field. In both cases, a few steps of the regularization produce an interface with a thickness of one cell.

The proposed regularization technique reduces the thickness of the interface and removes small spurious interface fragments. At the same time, it maintains exact volume conservation and boundness and does not affect the asymptotic convergence properties of the method as seen from Figs. S4, S6 and S7.

Time-reversed advection of circles

The following setup is based on a classical test case for advection schemes. The initial volume fraction field describes nine circles and the velocity field defined in a two-dimensional unit domain represents a single vortex reversed at time $t = 1.5$

$$\boldsymbol{u}(x, y, t) = \begin{cases} (\sin \pi x \, \cos \pi y, \, -\cos \pi x \, \sin \pi y), & t < 1.5\,, \\ (-\sin \pi x \, \cos \pi y, \, \cos \pi x \, \sin \pi y), & t \geq 1.5\,. \end{cases}$$

Since the advection equation is time-reversible, the exact solution at $t = 3$ coincides with the initial profile. Fig. S5 shows the solution in time and also compares the final profiles obtained by the standard PLIC advection and Multi-VOF on a mesh of N^2 cells for $N = 32$, 64, and 128. As seen from the final profiles at lower resolutions of $N = 32$ and 64, the standard advection method merges some of the circles into larger objects. This effect is the origin of numerical coalescence. The deformations reduce the distances between the interfaces, and eventually they merge since the method cannot describe multiple interfaces in the same cell. On the other hand, Multi-VOF correctly maintains all circles separated. Both methods converge to the same solution with mesh refinement.

Time-reversed advection of triple junction

The initial volume fraction field describes three components with a triple junction at the center. The velocity field defined in a two-dimensional unit domain $[0, 1]^2$ represents a single vortex reversed at time $t = 1$

$$\boldsymbol{u}(x, y, t) = \begin{cases} (\sin \pi x \, \cos \pi y, \, -\cos \pi x \, \sin \pi y), & t < 1\,, \\ (-\sin \pi x \, \cos \pi y, \, \cos \pi x \, \sin \pi y), & t \geq 1\,. \end{cases}$$

Fig. S6 shows the solution with Multi-VOF and the Hausdorff distance between interfaces computed on meshes of $(N/2)^2$ and N^2 cells for various N. The dependence of the mean Hausdorff distance on N indicates a first order convergence rate. Fig. S7 shows the same results without the interface regularization technique and confirms that it does not affect the convergence rate in the presence of multiple junctions.

Mean curvature flow with triple junctions

The following test case considers the evolution of multiple components with the velocity of the interface $\boldsymbol{u} = \kappa \boldsymbol{n}$ proportional to its curvature and normal. The discretization of this problem using the Multi-VOF method requires a divergence-free velocity field defined near the interface to maintain the volume of voids near triple junctions. Such a velocity field is found from $\boldsymbol{u} = \kappa \boldsymbol{n} - \nabla p$ and $\nabla \cdot \boldsymbol{u} = 0$ imposed in cells containing the interface fragments. In other cells, the scalar field p is set to zero. The initial configuration of $n + 1$ components in a two-dimensional unit domain describes a circle of a radius 0.3 connected to n lines at equal angles. The boundary conditions fix the positions and angles of the interfaces at the domain boundaries. From von Neumann-Mullins' law (*46, 47*), the exact rate of change of area $A(t)$ of the central component depends only on the number of triple junctions and equals $\frac{dA}{dt} = 2\pi(\frac{n}{6} - 1)$.

The solutions by Multi-VOF are compared with that of VIIM (*48*) on a uniform mesh of N^2 cells for $N = 32$, 64, 128, 256, and 512. The solution is advanced until time $T = 0.08$ and the time step is set to $\Delta t = 0.5h^2$ depending on the mesh step h. Results by VIIM include two cases depending on the value of parameter ϵ defining the distance between the interface and the level-set

inside each component: $\epsilon = 2h$ proportional to the mesh step h and a limiting case $\epsilon = 0^+$ which gives more accurate results but requires the use of one-sided finite differences that do not reach across the interfaces. Applications of VIIM presented in (*48*) use both values of this parameter.

Fig. S8 shows contour plots obtained using Multi-VOF with $N = 256$ and contours from VIIM at the same time instants. Plots of the error in $\frac{dA}{dt}$ show the absolute error $\left|\frac{dA}{dt} - 2\pi(\frac{n}{6} - 1)\right|$ where $\frac{dA}{dt}$ is the slope of the least-squares fit to $A(t)$. Plots of the mean Hausdorff distance show $\frac{1}{T}\int d_H(N, N/2; t)dt$, where $d_H(N, N/2; t)$ is the Hausdorff distance between interfaces at time t computed on meshes with $(N/2)^2$ and N^2 cells. The contour plots indicate a good agreement with VIIM in terms of the shapes of the interfaces. While the plots of the mean Hausdorff distance indicate a first order convergence rate, the error compared to the exact solution for the rate of change of area saturates at about 0.1 in cases $n = 4, 6$ and 0.2 in case $n = 8$. We attribute the lack of convergence to constant errors near triple junctions since their size relative to the cell size does not change with mesh refinement. Nevertheless, at lower resolutions the accuracy of our method is comparable to that of VIIM which operates exclusively in the dry foam regime while Multi-VOF also considers wet foams.

Buoyancy-driven breakup

This case shows a buoyancy-driven breakup of two bubbles on a free surface and serves as a convergence test of Multi-VOF coupled with the Navier-Stokes equations. The problem is solved in the two-dimensional unit domain $[0, 1]^2$ bounded by free-slip walls in the vertical direction and periodic in the horizontal direction. The mesh consists of N^2 cells for $N = 32$, 64, 128, 256, 512, and 1024. The initial velocity is zero, and the volume fraction field represents a layer of gas $0.6 < y < 1$, one bubble of a radius $R = 0.15$, and one bubble of a radius $R/2 = 0.075$. Parameters are the density $\rho_1 = 1$ and viscosity $\mu_1 = 0.005$ of the liquid, density and viscosity ratio $\rho_1/\rho_2 = \mu_1/\mu_2 = 100$, gravitational acceleration $g = 5$, and surface tension $\sigma = 0.01$. They correspond to $\mathrm{Eo} = \rho_1 g R^2/\sigma = 11.25$ and $\mathrm{Ga} = gR^3\rho_1^2/\mu_1^2 = 675$. One physical realization would be an air bubble of a radius 7.8 mm in an 80% aqueous solution of glycerol with density 1209 $\mathrm{kg/m^3}$, viscosity 0.1 $\mathrm{Pa \cdot s}$ and surface tension 0.065 N/m.

Fig. S9 presents the solution in time for various N and the corresponding Hausdorff distance between the interfaces computed on meshes with $(N/2)^2$ and N^2 cells. The bubbles rise and deform the free surface. The small bubble underneath penetrates the larger bubble and splits it into two equal parts. The breakup occurs at about $t = 0.6$ and manifests as an abrupt increase of the Hausdorff distance. In the final configuration, three bubbles rest on the surface. The dependence of the mean Hausdorff distance on N indicates a first order convergence rate.

To demonstrate that the method can handle higher density ratios, results for $\rho_1/\rho_2 = \mu_1/\mu_2 =$ 800 and 100 are compared in Fig. S10. The solutions are computed on a mesh of 256^2 cells. Increasing the density ratio has no effect before the breakup and causes a separation of the two smaller bubbles after the breakup. The time step in this case is set to $\Delta t = 2\sqrt{\frac{h^3\rho_2}{4\pi\sigma}}$.

Packing of rising bubbles

The case of close packing of rising bubbles demonstrates how overlapping interfaces are distributed over multiple layers. From this problem, we determine the number of layers L to use in other simulations and initially set it to $L = 8$. The problem is solved in the unit domain $[0, 1]^3$ on a mesh of 64^3 cells bounded by free-slip walls in the vertical direction and periodic in the other directions. The initial velocity is zero, and the volume fraction field represents a layer of gas

$0.8 < y < 1$ together with 397 bubbles of a radius 0.05 placed uniformly over the remaining volume. Parameters of the problem are the density $\rho_1 = 1$ and $\rho_2 = 0.01$, viscosity $\mu_1 = 0.01$ and $\mu_2 = 0.0001$, gravitational acceleration $g = 5$, and surface tension $\sigma = 0.1$.

Fig. S11 shows the snapshots from the simulation and the percentage of cells containing a given number of interfaces. The bubbles rise and closely pack creating bulges on the free surface. The initial separation between the bubbles is sufficiently large so that most cells contain only one interface. As the bubbles rise, the gaps between them reduce and the percentage of cells with multiple interfaces increases. At the final time $t = 10$, the percentages of cells containing from one to six interfaces amount respectively to 34.11%, 10.75%, 2.13%, 0.13%, 0.0034%, and 0.00076%. None of the cells contain more than six interfaces. Since the bubbles at the final time form a densely packed cluster and 99.9958% of cells contain four interfaces or fewer, we use $L = 4$ layers for all other simulations.

Drop impact on liquid-liquid interface

This test validates the model against experimental data on the gravity-driven impact of a liquid drop onto a liquid-liquid interface and shows that our method gives the same results as the multi-marker volume-of-fluid method (*19*). The problem is solved in a rectangular domain of size $5 \times 10 \times 5$ cm on a mesh containing $160 \times 320 \times 160$ cells bounded by no-slip walls in the vertical direction and periodic conditions in the other directions. Parameters of the problem are taken from numerical study (*19*) based on experiment (*49*) (Combination 1): density $\rho_1 = 949$ and $\rho_2 = 1128$ kg/m^3, viscosity $\mu_1 = 0.019$ and $\mu_2 = 0.0063$ Pa $\cdot$ s, gravitational acceleration $g = 9.8$ m/s^2, and surface tension coefficient $\sigma = 0.029$ N/m. They correspond to a glycerin-water drop falling in silicon oil. A spherical drop of a radius 5.1 mm is initially placed at a distance of 67 mm between its center and the interface.

Fig. S12 compares the cross sections of the interface obtained using Multi-VOF, numerical data (*19*), and experimental data (*49*). The falling drop approaches the surface separating the liquids, creates a bulge, and eventually rests on the surface. The liquid film between the liquids drains but no coalescence occurs. Our algorithm produces the same results as the method in (*19*) and both agree with the experimental data.

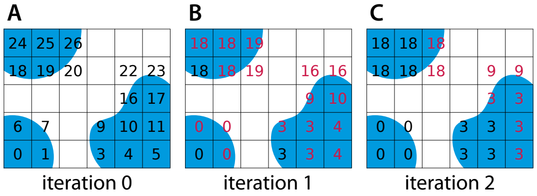

Fig. S1. Example of connected-component labeling. Mesh 6×5 mesh with a single layer $L = 1$. Current colors $\tilde{q}_c^l$ are shown with numbers. Initial colors (**A**) are indices enumerating all cells. Every iteration (**B**, **C**) selects the minimal color over the 3×3 stencil. Colors updated at the current iteration are highlighted in red.

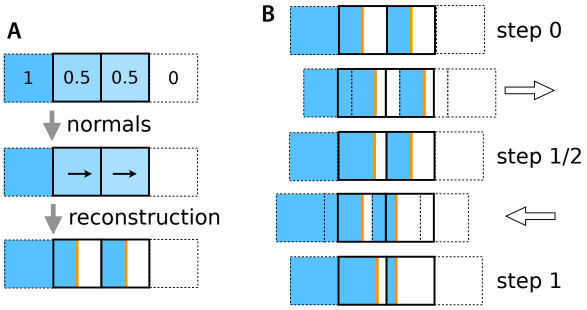

Fig. S2. Advection and interface regularization. (**A**) Stages of PLIC reconstruction in one dimension. The interface in each cell is replaced by a plane (orange) which is oriented by the estimated normal and cuts the cell at the given volume fraction. (**B**) Forward-backward advection reducing the interface thickness.

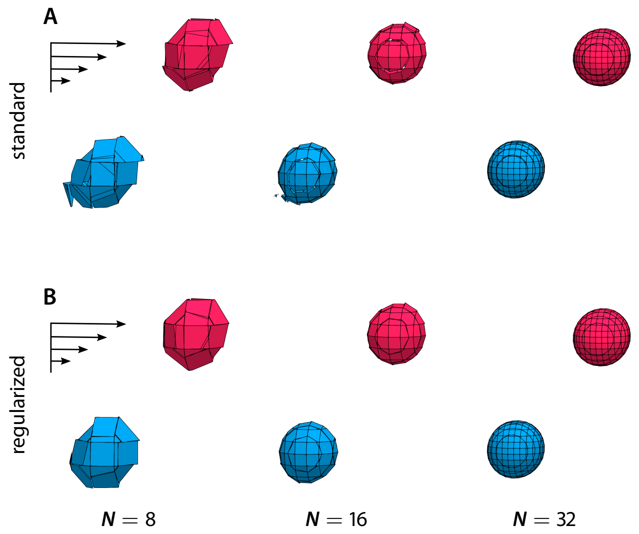

Fig. S3. Effect of interface regularization on drops in Couette flow. Simulations using advection without (**A**) and with interface regularization (**B**). Snapshots at $t = 0.75$ on a mesh of N^3 cells for $N = 8$, 16, and 32.

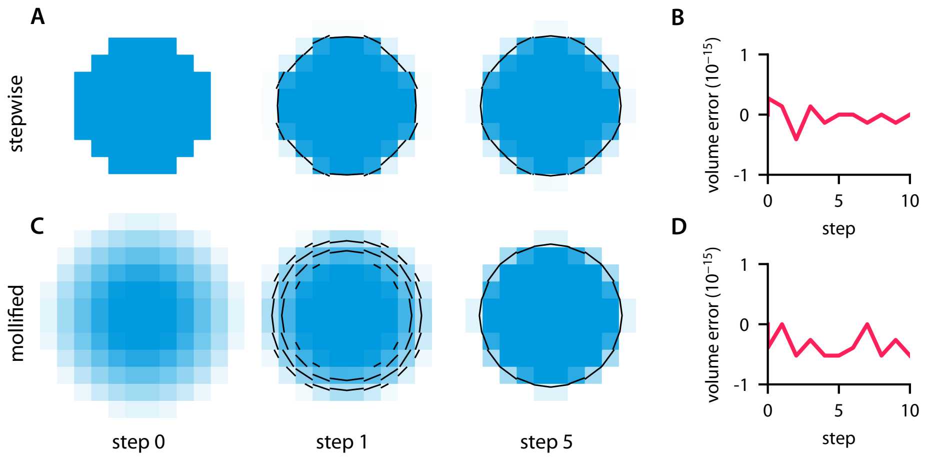

Fig. S4. Interface regularization applied to two fields representing a circle. Regularization steps starting from a stepwise (**A**) and radial piecewise linear (**C**) initial field. Relative error in the circle volume (**B**, **D**) shows that the interface regularization conserves volume up to machine precision.

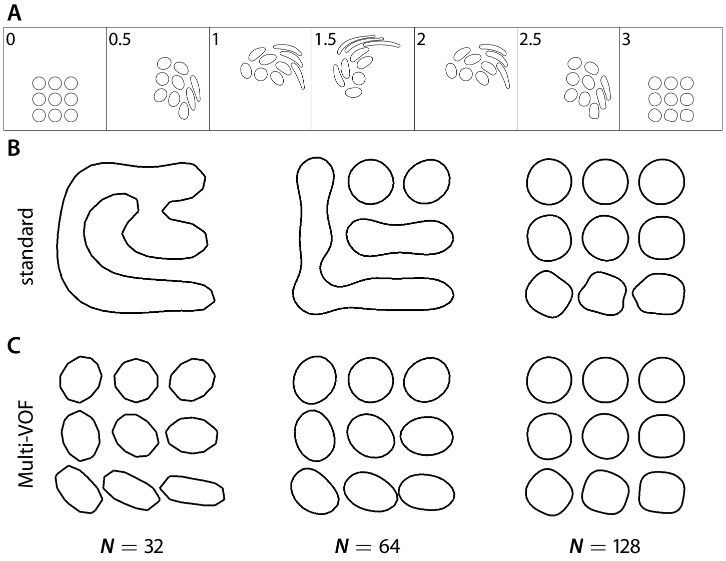

Fig. S5. Time-reversed advection of circles. (**A**) Snapshots at times t = 0, 0.5, 1, 1.5, 2, 2.5, and 3 on the finest mesh of 128^2 cells with Multi-VOF. (**B**, **C**) Final profiles at t = 3 with standard advection (**B**) and Multi-VOF (**C**) on a sequence of meshes of N^2 cells with N = 32, 64, and 128. The exact solution at t = 3 coincides with the initial profile.

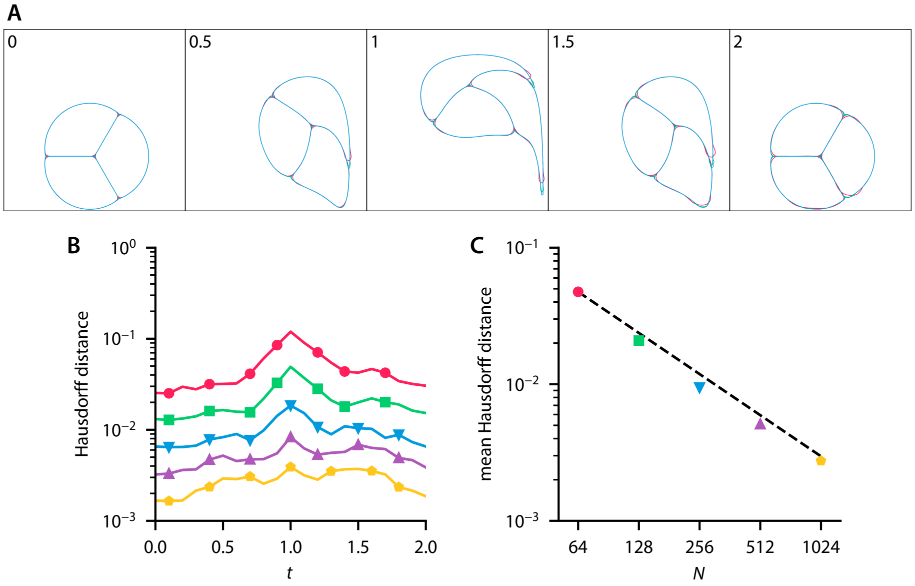

Fig. S6. Time-reversed advection of a triple junction. (**A**) Snapshots at times $t = 0, 0.5, 1, 1.5$, and 2 computed on a mesh of N^2 cells for $N = 64$ ——, 128 ——, and 256 ——. (**B**) Evolution of the Hausdorff distance between the interfaces computed on meshes with $(N/2)^2$ and N^2 cells for $N = 64$ —●—, 128 —■—, 256 —▼—, 512 —▲—, and 1024 —⬟—. (**C**) Mean Hausdorff distance over time versus the mesh size N. First order rate $O(1/N)$ - - -.

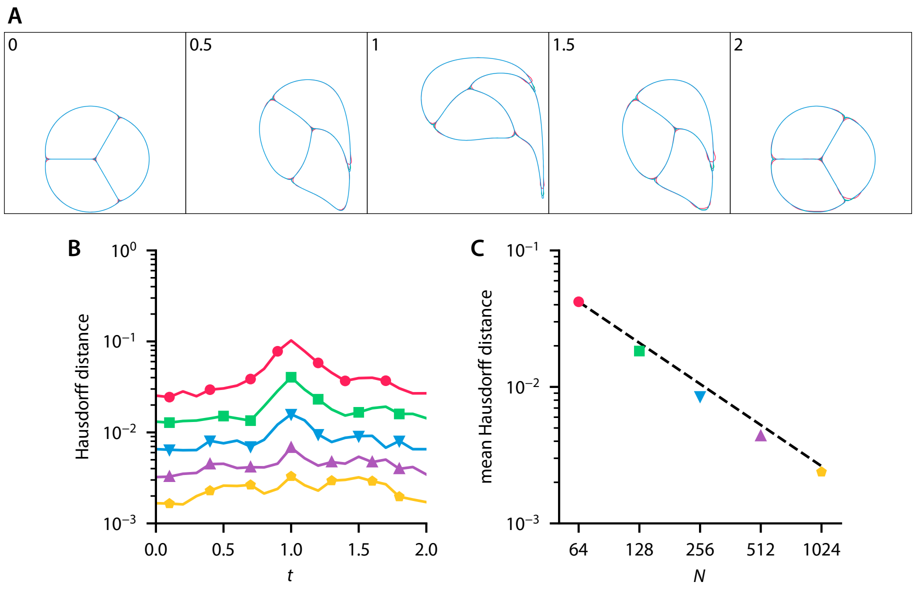

Fig. S7. Time-reversed advection of a triple junction without interface regularization. (**A**) Snapshots at times $t = 0, 0.5, 1, 1.5$, and 2 computed on a mesh of N^2 cells for $N = 64$ (red line), 128 (green line), and 256 (blue line). (**B**) Evolution of the Hausdorff distance between the interfaces computed on meshes with $(N/2)^2$ and N^2 cells for $N = 64$ (red, circles), 128 (green, squares), 256 (blue, downward triangles), 512 (purple, upward triangles), and 1024 (yellow, pentagons). (**C**) Mean Hausdorff distance over time versus the mesh size N. First order rate $O(1/N)$ (dashed line).

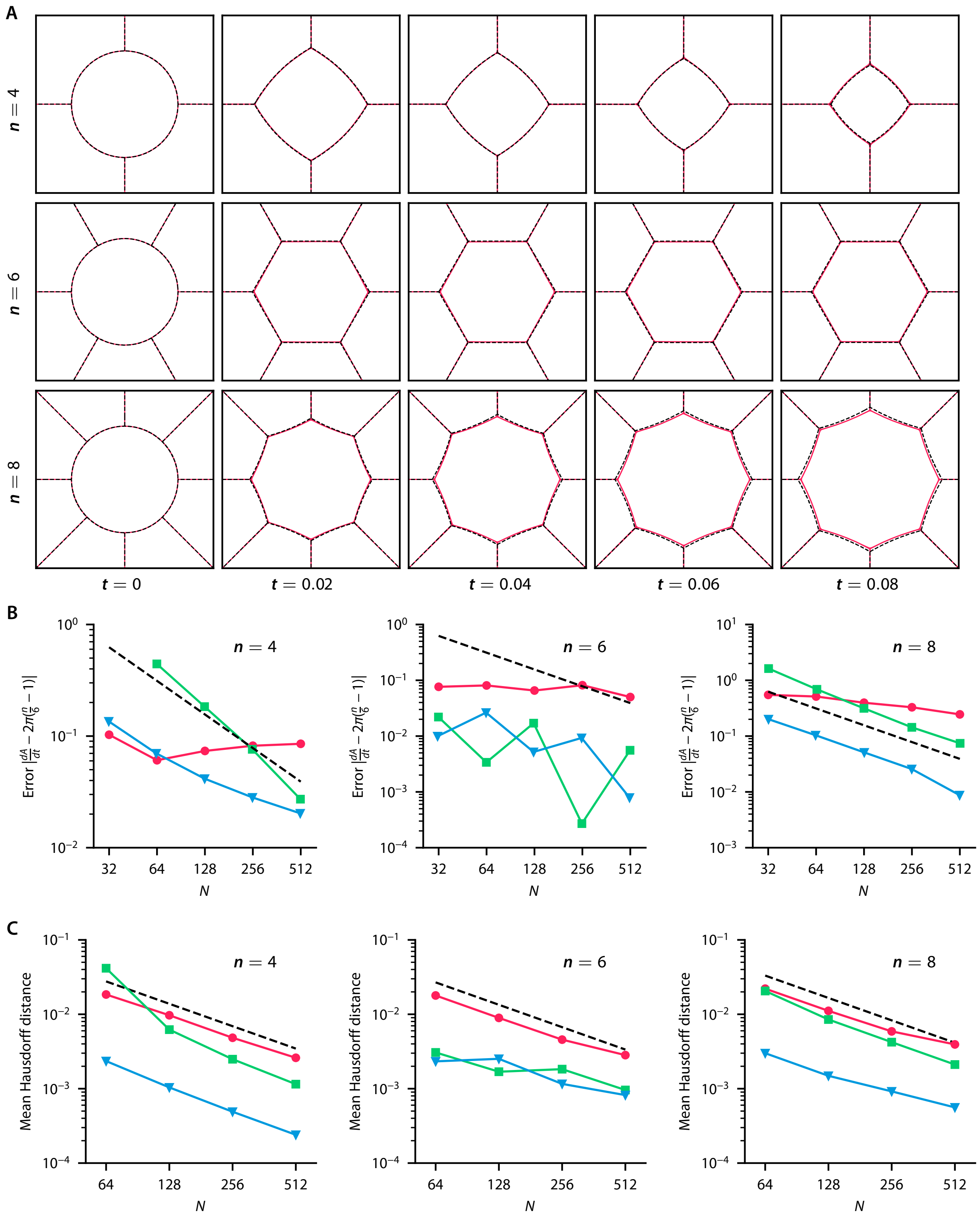

Fig. S8. Mean curvature flow with n triple junctions. (**A**) Interfaces at $t = 0, 0.02, 0.05, 0.06,$ and 0.08 computed using Multi-VOF and VIIM (*48*). (**B**) Absolute error $\left|\frac{dA}{dt} - 2\pi(\frac{n}{6} - 1)\right|$ in the area growth rate compared to von Neumann-Mullins's law using Multi-VOF, VIIM $\epsilon = 2h$, and VIIM $\epsilon = 0^+$. First order rate $\mathcal{O}(1/N)$. (**C**) Mean Hausdorff distance between interfaces computed using Multi-VOF, VIIM $\epsilon = 2h$, and VIIM $\epsilon = 0^+$ on meshes with $(N/2)^2$ and N^2 cells. First order rate $\mathcal{O}(1/N)$.

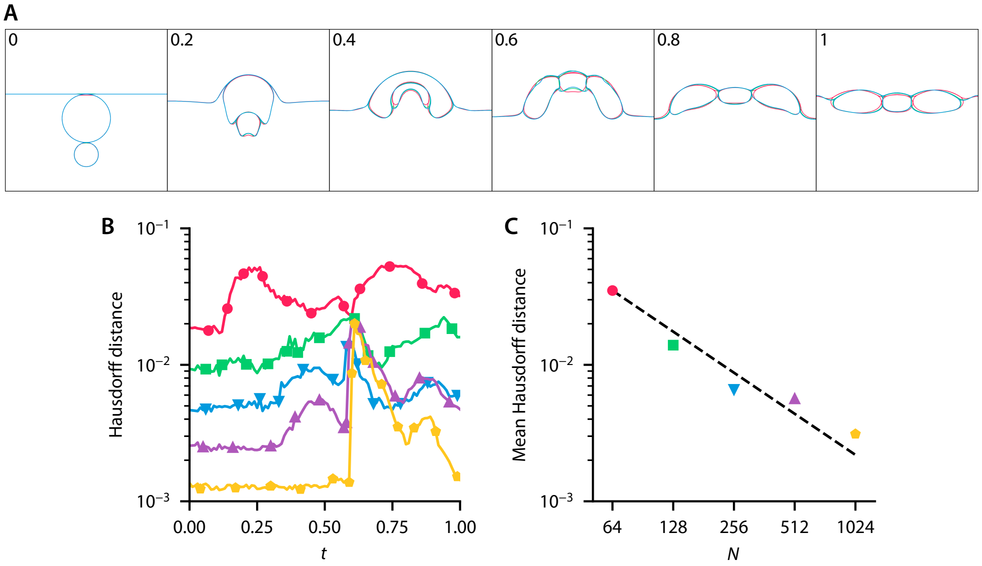

Fig. S9. Buoyancy-driven breakup of bubbles. (**A**) Snapshots at $t = 0, 0.2, 0.4, 0.6, 0.8$, and 1 computed on a mesh of N^2 cells for $N = 64$ ——, 128 ——, and 256 ——. (**B**) Evolution of the Hausdorff distance between the interfaces computed on meshes of $(N/2)^2$ and N^2 cells for $N = 64$ —●—, 128 —■—, 256 —▼—, 512 —▲—, and 1024 —⬟—. (**C**) Mean Hausdorff distance over time versus the mesh size N. First order rate $O(1/N)$ – – –.

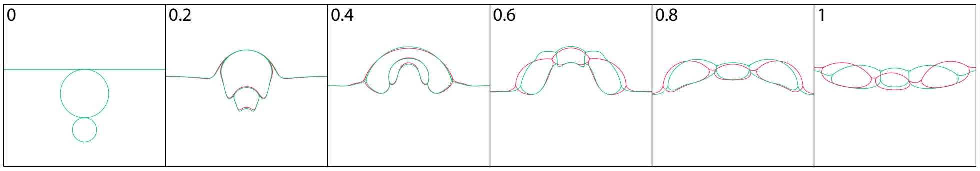

Fig. S10. Buoyancy-driven breakup of bubbles. Snapshots at $t = 0$, 0.2, 0.4, 0.6, 0.8, and 1 computed on a mesh of 256^2 cells with $\rho_1/\rho_2 = 800$ (red line) and 100 (green line).

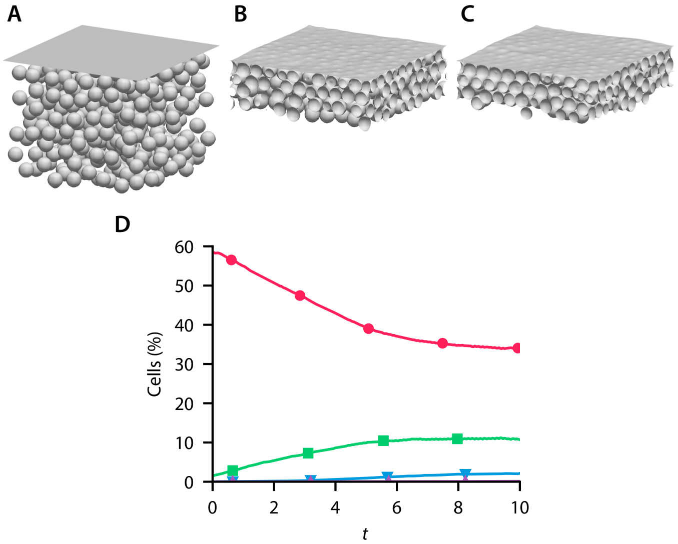

Fig. S11. Packing of rising bubbles. (**A-C**) Snapshots of the interfaces at $t = 0$, 5 and 10. (**D**) Percentage of cells with one, two, three, and four interfaces.

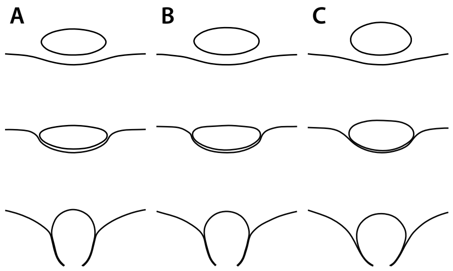

Fig. S12. Drop impact on liquid-liquid interface. Central cross sections at t = 0.63, 0.67, and 0.77 s produced by the present method (**A**) compared to images from numerical study (*19*) (**B**) and experimental data (*49*) (**C**).

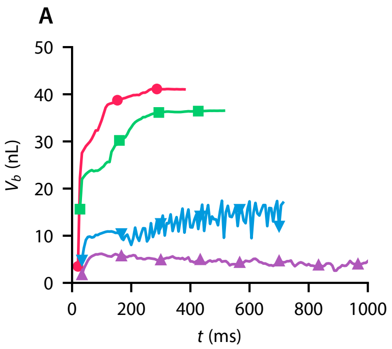

Fig. S13. Microfluidic crystals. Evolution of the volume of generated bubbles. (**A**) Stationary crystalline structures in channel of a length L = 6.8 mm. Hex-one (P_g = 216 Pa) —●—, hex-two (P_g = 194 Pa) —■—, hex-three (P_g = 149 Pa) —▼—, and hex-four (P_g = 140 Pa) —▲—. (**B**) Spontaneous transitions between hex-one and hex-two at P_g = 225 Pa and the channel length L = 5.3 mm.

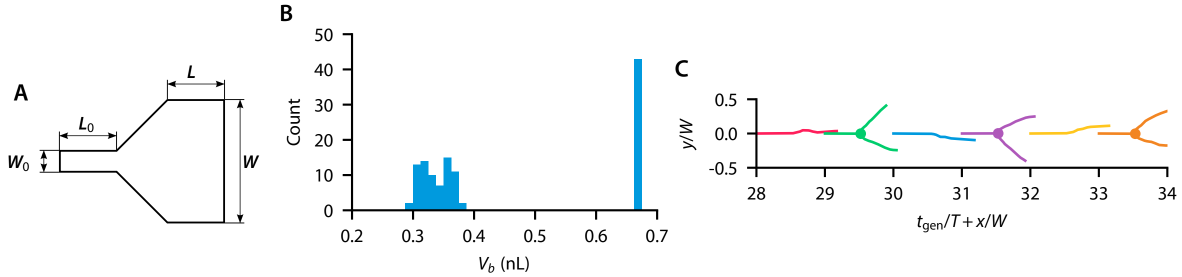

Fig. S14. Bidisperse foam generation. (**A**) Schematic of the device. (**B**) Histogram of the bubble volume after 80 cycles. (**C**) Trajectories of the centroids of bubbles generated at $t_{\mathrm{gen}}/T \in [28, 34)$ shifted along the horizontal axis by the generation time. Coordinates of the centroid x and y are relative to the generation point.

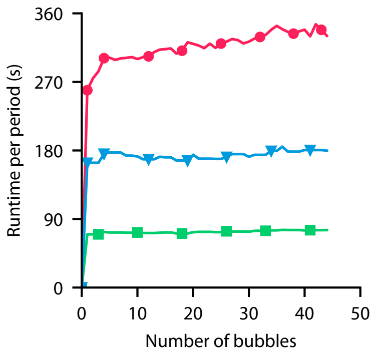

Fig. S15. Clustering of bubbles. Measured wall-clock time per bubble generation period versus the number of generated bubbles. Advection, Navier-Stokes, and total time including statistics and output. This shows that the cost of Multi-VOF does not depend on the number of bubbles.

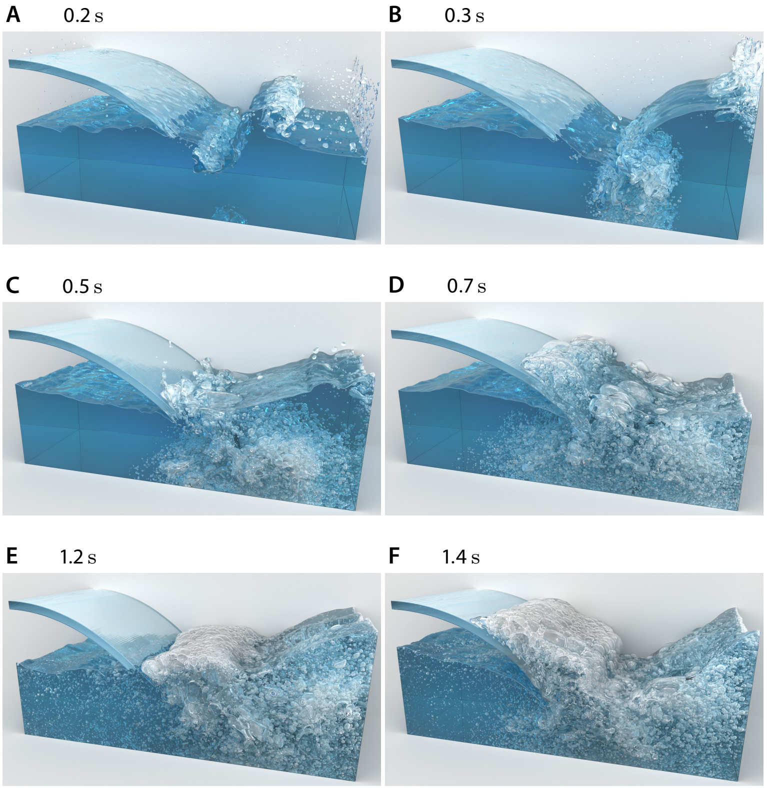

Fig. S16. Foaming waterfall. Snapshots of the interface at t = 0.2 (**A**), 0.3 (**B**), 0.5 (**C**), 0.7 (**D**), 1.2 (**E**), and 1.4 s (**F**).

density of liquid	ρ_l	1000	kg / m^3
density of gas	ρ_g	100	kg / m^3
viscosity of liquid	μ_l	0.25	mPa · s
viscosity of gas	μ_g	0.025	mPa · s
surface tension	σ	3.6	mN / m
liquid flow rate	Q_l	0.3	mL / h
bubble breakup period	T_b	$0.5\, W_0^2 H / Q_l$	
device height	H	100	μm
orifice width	W_0	110	μm
collection channel width	W	1000	μm
collection channel length	L	6800	μm
computational cell size	h	10.1	μm
Case of bubbling oscillator:			
collection channel length	L	5300	μm
bubble breakup period	T_b	$0.46\, W_0^2 H / Q_l$	
inlet pressure	P_g	225	Pa

Table S1. Microfluidic crystals. Simulation parameters.

density of liquid	ρ_l	1021	kg / m^3
density of gas	ρ_g	41	kg / m^3
viscosity of liquid	μ_l	1.25	mPa · s
viscosity of gas	μ_g	0.05	mPa · s
surface tension	σ	35.2	mN / m
bubble generation period	T	111	μs
volume of generated bubble	V_b	0.68	nL
gas volume fraction	Φ	0.645	–
inlet mean velocity	v_{mean}	2.09	m / s
inlet flow rate	Q	12	mL / h
channel height	H	60	μm
inlet channel width	W_0	75	μm
inlet channel length	L_0	816	μm
collection channel width	W	1600	μm
collection channel length	L	1600	μm
computational cell size	h	2.51	μm

Table S2. Bidisperse foam generation. Simulation parameters. Schematic in Fig. S14 illustrates the geometrical parameters.

density of liquid	1000	kg / m^3
density of gas	30	kg / m^3
viscosity of liquid	$2 \cdot 10^{-3}$	$Pa \cdot s$
viscosity of gas	$2 \cdot 10^{-5}$	$Pa \cdot s$
surface tension	18	mN / m
bubble diameter	2	mm
domain length	30	mm
domain width	30	mm
domain height	20	mm
gravitational acceleration	10	m/s^2
bubble generation period	0.05	s
computational cell size	0.16	mm

Table S3. Clustering of bubbles. Simulation parameters.

density of liquid	1000	kg / m^3
density of gas	10	kg / m^3
viscosity of liquid	10^{-3}	$Pa \cdot s$
viscosity of gas	10^{-5}	$Pa \cdot s$
surface tension	72	mN / m
waterfall mean velocity	1.5	m/s
waterfall thickness	5	mm
domain length	200	mm
domain width	100	mm
domain height	100	mm

Table S4. Foaming waterfall. Simulation parameters.

Application	Mesh	Simulation time	CPU cores	Time steps	Wall-clock time
Microfluidic crystals	$792 \times 104 \times 16$	1000 ms	234	137000	24 hours
Bidisperse foam generation	$1280 \times 656 \times 32$	9000 μs	1640	50000	24 hours
Clustering of bubbles	$192 \times 128 \times 192$	3.7 s	1152	57000	10 hours
Foaming waterfall	$768 \times 384 \times 384$	1.4 s	13824	47000	24 hours

Table S5. Problem sizes and measured wall-clock time. Results of simulations on Piz Daint supercomputer equipped with 12-core Intel Xeon E5-2690 v3 processors.

Movie S1

Microfluidic crystal, hex-one.

Movie S2

Microfluidic crystal, hex-two.

Movie S3

Microfluidic crystal, hex-three.

Movie S4

Microfluidic crystal, hex-four.

Movie S5

Microfluidic crystal, oscillations between hex-one and hex-two.

Movie S6

Bidisperse foam generation.

Movie S7

Clustering of bubbles floating in water.

Movie S8

Foaming waterfall.

REFERENCES AND NOTES

1. M. Stoffel, S. Wahl, E. Lorenceau, R. Hoehler, B. Mercier, D. E. Angelescu, Bubble production mechanism in a microfluidic foam generator. *Phys. Rev. Lett.* **108**, 198302 (2012).

2. S. L. Anna, Droplets and bubbles in microfluidic devices. *Annu. Rev. Fluid Mech.* **48**, 285–309 (2016).

3. A. Prosperetti, Vapor bubbles. *Annu. Rev. Fluid Mech.* **49**, 221–248 (2017).

4. L. A. Del Castillo, S. Ohnishi, R. G. Horn, Inhibition of bubble coalescence: Effects of salt concentration and speed of approach. *J. Colloid Interface Sci.* **356**, 316–324 (2011).

5. V. Craig, B. Ninham, R. Pashley, Effect of electrolytes on bubble coalescence. *Nature* **364**, 317–319 (1993).

6. D. Y. Chan, E. Klaseboer, R. Manica, Film drainage and coalescence between deformable drops and bubbles. *Soft Matter* **7**, 2235–2264 (2011).

7. H. A. Stone, Tuned-in flow control. *Nat. Phys.* **5**, 178–179 (2009).

8. C. Hill, J. Eastoe, Foams: From nature to industry. *Adv. Colloid Interf. Sci.* **247**, 496–513 (2017). Dominique Langevin Festschrift: Four decades opening gates in colloid and interface science.

9. B. Dollet, P. Marmottant, V. Garbin, Bubble dynamics in soft and biological matter. *Annu. Rev. Fluid Mech.* **51**, 331–355 (2019).

10. G. Tryggvason, S. Thomas, J. Lu, B. Aboulhasanzadeh, Multiscale issues in DNS of multiphase flows. *Acta Math. Sci.* **30**, 551–562 (2010). Dedicated to professor James Glimm on the occasion of his 75th birthday.

11. S. Cohen-Addad, R. Höhler, O. Pitois, Flow in foams and flowing foams. *Annu. Rev. Fluid Mech.* **45**, 241–267 (2013).

12. K. A. Brakke, The surface evolver. *Exp. Math.* **1**, 141–165 (1992).

13. R. I. Saye, J. A. Sethian, The Voronoi implicit interface method for computing multiphase physics. *Proc. Natl. Acad. Sci.* **108**, 19498–19503 (2011).

14. R. I. Saye, J. A. Sethian, Multiscale modeling of membrane rearrangement, drainage, and rupture in evolving foams. *Science* **340**, 720–724 (2013).

15. A. Montessori, A. Tiribocchi, F. Bonaccorso, M. Lauricella, S. Succi, Lattice Boltzmann simulations capture the multiscale physics of soft flowing crystals. *Phil. Trans. R. Soc. A* **378**, 20190406 (2020).

16. J. Fang, M. Rasquin, I. A. Bolotnov, Interface tracking simulations of bubbly flows in PWR relevant geometries. *Nucl. Eng. Des.* **312**, 205–213 (2017).

17. C. Hirt, B. Nichols, Volume of fluid (VOF) method for the dynamics of free boundaries. *J. Comput. Phys.* **39**, 201–225 (1981).

18. A. Prosperetti, G. Tryggvason, *Computational Methods for Multiphase Flow* (Cambridge Univ. Press, 2009).

19. E. Coyajee, B. J. Boersma, Numerical simulation of drop impact on a liquid–liquid interface with a multiple marker front-capturing method. *J. Comput. Phys.* **228**, 4444–4467 (2009).

20. N. Balcázar, O. Lehmkuhl, J. Rigola, A. Oliva, A multiple marker level-set method for simulation of deformable fluid particles. *Int. J. Multiphase Flow* **74**, 125–142 (2015).

21. M. Kwakkel, W.-P. Breugem, B. J. Boersma, An efficient multiple marker front-capturing method for two-phase flows. *Comput. Fluids* **63**, 47–56 (2012).

22. D. Anderl, S. Bogner, C. Rauh, U. Rüde, A. Delgado, Free surface lattice Boltzmann with enhanced bubble model. *Comput. Math. Appl.* **67**, 331–339 (2014).

23. T. J. Spencer, I. Halliday, C. M. Care, A local lattice Boltzmann method for multiple immiscible fluids and dense suspensions of drops. *Philos. Trans. R. Soc. A Math. Phys. Eng. Sci.* **369**, 2255–2263 (2011).

24. T. Beatus, T. Tlusty, R. Bar-Ziv, Phonons in a one-dimensional microfluidic crystal. *Nat. Phys.* **2**, 743–748 (2006).

25. P. Marmottant, J.-P. Raven, Microfluidics with foams. *Soft Matter* **5**, 3385–3388 (2009).

26. J.-P. Raven, P. Marmottant, Microfluidic crystals: Dynamic interplay between rearrangement waves and flow. *Phys. Rev. Lett.* **102**, 084501 (2009).

27. P. Garstecki, H. A. Stone, G. M. Whitesides, Mechanism for flow-rate controlled breakup in confined geometries: A route to monodisperse emulsions. *Phys. Rev. Lett.* **94**, 164501 (2005).

28. I. Cantat, N. Kern, R. Delannay, Dissipation in foam flowing through narrow channels. *Europhys. Lett.* **65**, 726–732 (2004).

29. J.-P. Raven, P. Marmottant, Periodic microfluidic bubbling oscillator: Insight into the stability of two-phase microflows. *Phys. Rev. Lett.* **97**, 154501 (2006).

30. D. Vecchiolla, V. Giri, S. L. Biswal, Bubble–bubble pinch-off in symmetric and asymmetric microfluidic expansion channels for ordered foam generation. *Soft Matter* **14**, 9312–9325 (2018).

31. D. Vella, L. Mahadevan, The "Cheerios effect". *Am. J. Phys.* **73**, 817–825 (2005).

32. K. T. Kiger, J. H. Duncan, Air-entrainment mechanisms in plunging jets and breaking waves. *Annu. Rev. Fluid Mech.* **44**, 563–596 (2012).

33. C. Garrett, M. Li, D. Farmer, The connection between bubble size spectra and energy dissipation rates in the upper ocean. *J. Phys. Oceanogr.* **30**, 2163–2171 (2000).

34. G. B. Deane, M. D. Stokes, Scale dependence of bubble creation mechanisms in breaking waves. *Nature* **418**, 839–844 (2002).

35. L. Deike, W. K. Melville, S. Popinet, Air entrainment and bubble statistics in breaking waves. *J. Fluid Mech.* **801**, 91–129 (2016).

36. G.-H. Cottet, E. Maitre, A semi-implicit level set method for multiphase flows and fluid–structure interaction problems. *J. Comput. Phys.* **314**, 80–92 (2016).

37. S. L. Anna, N. Bontoux, H. A. Stone, Formation of dispersions using flow focusing in microchannels. *Appl. Phys. Lett.* **82**, 364–366 (2003).

38. A. Mata, A. J. Fleischman, S. Roy, Characterization of polydimethylsiloxane (PDMS) properties for biomedical micro/nanosystems. *Biomed. Microdevices* **7**, 281–293 (2005).

39. D. Youngs, Time-dependent multi-material flow with large fluid distortion, in *Numerical Methods in Fluid Dynamics* , K. W. Morton, M. J. Baines, Eds. (Academic Press, New York, 1982), vol. 24, pp. 273–285.

40. E. Aulisa, S. Manservisi, R. Scardovelli, S. Zaleski, Interface reconstruction with least-squares fit and split advection in three-dimensional cartesian geometry. *J. Comput. Phys.* **225**, 2301–2319 (2007).

41. R. Scardovelli, S. Zaleski, Analytical relations connecting linear interfaces and volume fractions in rectangular grids. *J. Comput. Phys.* **164**, 228–237 (2000).

42. G. D. Weymouth, D. K.-P. Yue, Conservative volume-of-fluid method for free-surface simulations on cartesian-grids. *J. Comput. Phys.* **229**, 2853–2865 (2010).

43. J. B. Bell, P. Colella, H. M. Glaz, A second-order projection method for the incompressible Navier-Stokes equations. *J. Comput. Phys.* **85**, 257–283 (1989).

44. P. Karnakov, S. Litvinov, P. Koumoutsakos, A hybrid particle volume-of-fluid method for curvature estimation in multiphase flows. *Int. J. Multiphase Flow* **125**, 103209 (2020).

45. P. Colella, D. T. Graves, B. J. Keen, D. Modiano, A Cartesian grid embedded boundary method for hyperbolic conservation laws. *J. Comput. Phys.* **211**, 347–366 (2006).

46. W. W. Mullins, Two-dimensional motion of idealized grain boundaries. *J. Appl. Phys.* **27**, 900–904 (1956).

47. J. Von Neumann, Metal interfaces. *American Society for Metals, Cleveland* **108**, (1952).

48. R. I. Saye, J. A. Sethian, Analysis and applications of the Voronoi implicit interface method. *J. Comput. Phys.* **231**, 6051–6085 (2012).

49. Z. Mohamed-Kassim, E. K. Longmire, Drop impact on a liquid–liquid interface. *Phys. Fluids* **15**, 3263–3273 (2003).
